# Virtual Embodiment of White People in a Black Virtual Body Leads to a Sustained Reduction in Their Implicit Racial Bias

**DOI:** 10.3389/fnhum.2016.00601

**Published:** 2016-11-29

**Authors:** Domna Banakou, Parasuram D. Hanumanthu, Mel Slater

**Affiliations:** ^1^Event Lab, Department of Clinical Psychology and Psychobiology, University of BarcelonaBarcelona, Spain; ^2^Institute of Neurosciences, University of BarcelonaBarcelona, Spain; ^3^Department of Computer Science, University College LondonLondon, UK; ^4^Institució Catalana de Recerca i Estudis AvançatsBarcelona, Spain

**Keywords:** racial bias, racism, body ownership, virtual reality, implicit association test, rubber hand illusion, Tai Chi

## Abstract

Virtual reality can be used to visually substitute a person's body by a life-sized virtual one. Such embodiment results in a perceptual illusion of body ownership over the virtual body (VB). Previous research has shown that the form of the VB can influence implicit attitudes. In particular, embodying White people in a Black virtual body is associated with an immediate decrease in their implicit racial bias against Black people. We tested whether the reduction in implicit bias lasts for at least 1 week and whether it is enhanced by multiple exposures. Two experiments were carried out with a total of 90 female participants where the virtual body was either Black or White. Participants were required to follow a virtual Tai Chi teacher who was either Asian or European Caucasian. Each participant had 1, 2, or 3 exposures separated by days. Implicit racial bias was measured 1 week before their first exposure and 1 week after their last. The results show that implicit bias decreased more for those with the Black virtual body than the White. There was also some evidence of a general decrease in bias independently of body type for which possible explanations are put forward.

## Introduction

There are many interventions discussed in the literature designed to have the effect of reducing implicit racial bias of “White” people toward “Black” people. Seventeen interventions were described and evaluated by Lai et al. (2014) of which the most successful were those that involved examples that ran against stereotypical behavior, and the least effective involved perspective taking or invoking egalitarian values. However, in a subsequent study Lai et al. (2016) found that no intervention was successful in inducing a sustained reduction of racial bias whether for hours or days. In this paper we show how the technique of virtual embodiment, where a light-skinned person's body is visually substituted in immersive virtual reality by a life-sized spatially coincident dark-skinned virtual body, results in a reduction in implicit bias that lasts at least 1 week. The results provide a replication of earlier results described by Peck et al. (2013) and extend those results by considering whether the reduction of bias is sustained, and whether there is any effect of multiple exposures.

Several studies have shown that when people are virtually embodied or represented online with a virtual body different to their own then they exhibit behaviors concomitant with attributes of that body. Yee and Bailenson (2007) referred to this as the Proteus Effect. They reported, for example, that when participants have a virtual body that has a more attractive face than their real one their proxemics behavior alters—they stand closer to virtual representations of other people than when the virtual face is less attractive. People will become more aggressive in negotiation with others if embodied in a virtual body that is taller than if shorter (Yee and Bailenson, 2007). They also showed that when a White person is put into a scenario of a job interview but embodied in a Black virtual body they will later exhibit greater implicit racial bias against Black people than if in a White virtual body (Groom et al., 2009).

The Proteus Effect is premised on Self-Perception Theory (Bem, 1972). This argues that people observe their own behaviors in the given context and infer their attitudes based on what they themselves do in that situation. It has also been argued that stereotyping plays a role in this, where people behave as others would expect someone with that type of body to behave (Yee and Bailenson, 2007). For example, in the case of racial bias as reported by Groom et al. (2009) participants were placed in a job interview, which is a situation where implicit racial bias typically operates.

However, this approach cannot explain how there can be perceptual and implicit attitude changes when participants are embodied in a virtual body in a neutral situation—i.e., where there is no social context or when the effect is physiological or perceptual rather than social. For example, recent research employing multisensory integration techniques derived from the rubber hand illusion (Botvinick and Cohen, 1998) has shown that even pain and temperature sensitivity thresholds of people can be changed through transformed body ownership (Llobera et al., 2013; Martini et al., 2013). As another example where there was no social context, Banakou et al. (2013) carried out an experiment where adults were embodied in a virtual body representing a toddler (4 or 5 years old), or an adult body that was shrunk down to the size of the child, so that the eye heights were the same in both cases. Hence the only difference between the two bodies was the body shape—childlike or adult-like. When the virtual body moved synchronously with the real body movements of the participant then there was strong body ownership over the virtual body—irrespective as to whether it was the child or adult one. Moreover, participants with the child body approximately doubly overestimated the sizes of objects in the virtual environment, comparing after with before the stimulation, whereas those with the adult body did not. An Implicit Association Test (IAT) (Greenwald et al., 1998) was used to test the extent to which participants associated child-like or adult like attributes to themselves. Those in the child body condition associated child-like attributes to themselves far more than those with the adult body (who hardly made such attributes at all). When there was visuomotor asynchrony (the virtual body moved independently of the real one, even though there was still first person perspective view over the body) all of these results were extinguished. This was in a context where the only task of participants was to look at themselves (seeing their virtual body) both directly looking down toward their real body, and in the virtual mirror. Hence in this case, since there was no social context, Self-Perception Theory and stereotyping could not apply.

It is possible that in the case of child embodiment the brain relies on autobiographical memory—since all the adults of course had once been children. In order to test this we turned to another example of embodiment where it would not be possible for autobiographical memory to be a factor. Peck et al. (2013) embodied White participants in a Black body. This was a between groups experiment (*n* = 15 per group), where White (female) participants were embodied in a Black body, a White body, a Purple body, or No Body. Participants saw their virtual body either directly by looking down toward themselves and in a virtual mirror. In the case of the “No body” condition participants saw a reflected Black body at the correct place geometrically in the mirror, but it moved asynchronously with their movements. In all other cases there was visuomotor synchrony. The scenario was neutral—participants spent altogether 12 min embodied in this way, in an empty room, and eventually 12 virtual characters walked by them (6 White characters and 6 Black). The characters entered their personal space as they walked by. Unlike the study of Groom et al. (2009) there was no social meaning to this that had anything in itself to do with racial bias. A racial IAT was used as the main response variable. It was administered approximately 3 days before the virtual reality exposure (preIAT) and then immediately after (postIAT). The variable of interest was dIAT = postIAT–preIAT.

The levels of body ownership were equivalent for the three embodied conditions and lower for the No body condition. Those with the Black body showed significantly less implicit racial bias after the experiment compared to before—in fact the mean level of dIAT < 0 only for this group. The inclusion of the purple body as a control showed that this was probably a racial effect rather than only being a difference or strangeness effect.

This result seemed to be stunning in the sense that it was hard to believe that 12 min of exposure to being embodied in a Black virtual body might alter something that is as apparently ingrained as implicit racial bias. However, similar results were reported by Maister et al. (2013) where the Rubber Hand Illusion (Botvinick and Cohen, 1998) with a black arm or white arm (control) was used. An overview of this set of experiments can be found in Maister et al. (2015).

In this paper we describe an experiment that takes these results further. Here the hypothesis was that embodiment of White people in dark-skinned virtual body would lead to a reduction in negative implicit racial bias toward Black people, as described earlier except that we were specifically interested in whether the illusion would last at least 1 week after the final exposure. We were also interested in whether multiple exposures might further strengthen the reduction in racial bias. We carried out two conceptually distinct experiments that we refer to as Experiment 1 and Experiment 2 in order to address these questions.

## Materials and methods

### Experimental design

This experiment was designed to address the question as to whether there is any sustained reduction in implicit racial bias—in particular that lasts at least 1 week -after embodiment of White participants in a Black body, and also whether the effect is influenced by the number of exposures.

**Figure 1:**
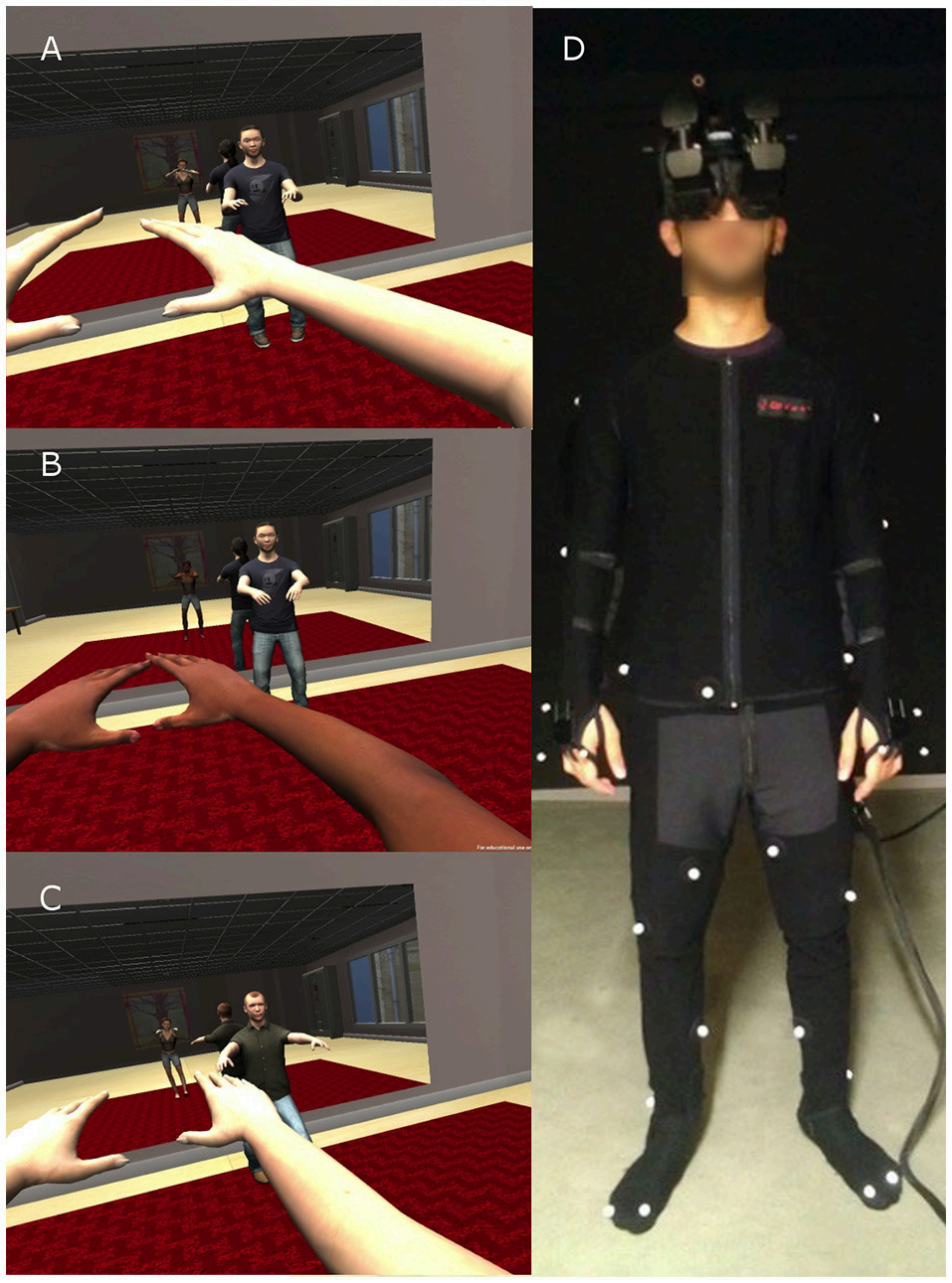
**The experimental scenario, with variations in the virtual body of the participant and the teacher. (A)** The participant is embodied in a White virtual body and the Teacher is Asian. **(B)** The participant is in a Black virtual body and the Teacher is Asian. **(C)** The participant is in a White virtual body and the teacher is European (Experiment 2). **(D)** The physical apparatus worn by participants—the head-mounted display and the motion capture suit.

The scenario was one where participants were encouraged to follow the movements of a (virtual) Tai Chi teacher. Each participant was embodied in either a Black or White body (Figures 1A,B). The participants had either 1, 2, or 3 exposures, each separate by 2 days. In Experiment 1 the Tai Chi teacher was of Asian appearance (Figures 1A,B). There was visuomotor synchrony so that through real-time motion capture the virtual body moved in synchrony and correspondence with real body movements. Each exposure consisted of a 5-min orientation period, and then the Embodiment condition for 10 min. The experiment is illustrated in Supplementary Video S1^1^.

In all conditions the participants were administered a racial IAT test 1 week before their first (or only) exposure (preIAT). They were administered a second IAT always 1 week after their final exposure (postIAT). Hence for each participant, no matter how many exposures they had, they were administered the IAT only twice—1 week before their first exposure and 1 week after their last exposure. The response variable of interest is dIAT = postIAT–preIAT.

Experiment 1 therefore followed a single factor between groups design and an independent variable. The factor was Embodiment (White, Black) indicating the type of body in which participants were embodied. The independent variable was Exposure (the number of exposures to the scenario, 1, 2, or 3). There were 10 participants per group, thus 60 in all, females recruited from the campus at the University of Barcelona.

Experiment 2 was carried out under the same conditions as the first and with another group of 30 participants. The difference was that the Teacher was of European appearance, and the participants (10 per cell) were always embodied as White. Hence the experimental independent variable was Exposures (1, 2, or 3 as before). The reason for this second experiment is explained in the Results section.

### Technical details

#### Environment

The experiment was conducted in a Virtual Reality (VR) lab (width: 2.96 m, length: 3.4 m—back wall to curtain—height: 2.87 m). Participants were fitted with a stereo NVIS nVisor SX111 head-mounted display. This has dual SXGA displays with 76°H × 64°V (degrees) field of view (FOV) per eye, totaling a wide field-of-view 111° horizontal with 50° (66%) overlap and 64° vertical, with a resolution of 1280 × 1024 pixels per eye displayed at 60 Hz. Head tracking was performed by a 6-DOF Intersense IS-900 device. Participants were also required to wear an Optitrack full body motion capture suit that uses 37 markers and the Motive software^2^ to track their movements (Figure 1D). The infrared technology was implemented with a 12-camera truss setup by OptiTrack. Virtual models created with 3D Studio Max 2010^3^ and Autodesk Character Generator^4^. The virtual environment was implemented on the Unity 3D platform^5^.

#### Tai Chi animation

The Tai Chi animation was based on a motion captured Tai Chi animation^6^, which was used as a reference to manually build the animation clips. As Tai Chi movements are challenging for beginners to perform, the continuous form of the original clip was broken down into shorter animation clips. These were triggered by the experimenter throughout the experiment. Nine short clips were used in total. The animation was implemented in Autodesk Motion Builder 2014^7^.

### Participants

Sixty (Experiment 1) and 30 (Experiment 2) female participants aged 18–44 years (mean age 21.9, *SD* = 4.45, *SE* = 0.47) with normal or corrected vision took part in this study. They had no prior knowledge of the experiment, and little or no prior experience of VR. Participants were recruited through posters, e-mail and word of mouth around the campus of the University of Barcelona. Preliminary checks established whether participants fitted the inclusion criteria (Caucasian only, no psychoactive medication, no mental-health disorders, no epilepsy). Participants were provided with an information sheet regarding issues such as the limits of confidentiality, right to withdraw (either during the experiment or later) etc. Written informed consent was obtained. The experimental groups were comparable across a number of variables. The age distribution of participants is shown per condition in Table 1. Almost all the participants were novices in computer programming. In Experiment 1 43/60 played computer video games to a moderate extent (47/60 less than twice a year) with little variation between the experimental groups. In Experiment 2, 24/30 played less than twice a year. On a scale of 1 (no experience) to 7 (extensive) previous experience of VR, the medians are at most 4 across all experimental groups, with the maximum IQR of 4.

**Table 1:** **Mean ± Standard Deviation of Age by experimental conditions**.

**Teacher Asian**	**Exposures**		
**Embodiment**	1	2	3
Black	21.5 ± 3.54	20.3 ± 1.41	22.4 ± 2.80
White	19.9 ± 1.73	20.8 ± 2.74	21.7 ± 2.36
**Teacher European**	26.9 ± 9.78	21.9 ± 3.25	22.0 ± 3.80

### Procedures

On a first visit, participants were given the study information sheet to read and after they agreed to participate they were given a consent form to sign. Next they completed a short demographics questionnaire (i.e., age, occupation, VR, and games experience etc.) and then completed a racial bias Implicit Association Test (IAT) (Greenwald et al., 1998, 2003) on a desktop computer, and the results were recorded (preIAT). After a period of 1 week they returned for their first (or only) exposure in the main experiment.

The position of all participants was controlled through Velcro strips on the floor that were used to mark where they should stand during the experiment. These positions corresponded to the centers of the physical and virtual room. Participants were instructed to turn and move their heads and bodies and walk a maximum two steps away from that area, to prevent them from hitting the walls due to the restricted laboratory space.

During the orientation phase of the experiment, participants entered a virtual room decorated with everyday furniture and a virtual mirror. The body of the participant was substituted by the Black or White virtual body, seen from first-person perspective (1PP) (Figure 1). The participant's head and body movements were mapped in real time to the virtual body. They could see this body both by looking directly toward their real body, and also in the virtual mirror. A series of instructions were then given to the participants from a pre-recorded audio. First, they were instructed to perform a simple set of stretching exercises in order to explore the capabilities and real time motion of the virtual body, including movements of their arms, legs and feet. They were asked to continue performing these exercises by themselves and also look around the virtual room in all directions, where they were asked to state and describe what they saw. After this 5-min orientation period, participants were instructed that the second virtual character they would see in the room in front of them would be a Tai Chi teacher who would perform different movements that they should follow. They were free to choose whether they performed each movement simultaneously with the teacher or after the movement was completed. The Tai Chi training went on for 10 min, the same for all conditions and exposure repetitions. The reason of choosing such a setup was to engage participants for the total time required in the virtual environment, and to constantly reinforce visuomotor synchrony. The whole procedure lasted approximately 35 min. Finally, the HMD was removed, and they completed a questionnaire about their experience, including questions about the level of subjective body ownership (Table 2). Those in the multiple exposure groups returned to the laboratory for their second and third sessions 2 days and 4 days later. One week after completion of the final exposure, all participants returned to the lab in order to perform the IAT again (postIAT). Two experimental operators (females) were present throughout the whole experiment.

**Table 2:** **Post Exposure Questionnaire—set of statements each on a −3 to 3 scale with −3 being strong disagreement and 3 being complete agreement**.

**Variable Name**	**Statement**
MyBody	“I felt that the virtual body I saw when looking down at myself was my own body”
TwoBodies	“I felt as if I had two bodies”
Mirror	“I felt that the virtual body I saw when looking at myself in the mirror was my own body”
Features	“I felt that my virtual body resembled my own (real) body in terms of shape, skin tone, or other visual features”
Agency	“I felt that the movements of the virtual body were caused by my own movements”

### Response variables

#### Racial implicit association test (IAT)

Implicit racial bias was calculated by administering participants an IAT (Greenwald et al., 1998), 1 week before their first virtual exposure, and 1 week after their last (or unique) virtual exposure. The IAT was completed on the same desktop computer screen both times.

The racial IAT followed the standard IAT procedure (Nosek et al., 2005), where participants are required to rapidly categorize faces (White or Black) and words (positive or negative) into groups. Implicit bias is calculated from the differences in accuracy and speed between categorizations (e.g., white faces, positive words and black faces, negative words compared to the opposite groups). Higher IAT scores are interpreted as the greater implicit racial bias, as this signifies longer reaction times and greater inaccuracies in categorizing black faces with positive words, and white faces with negative words. It has been shown that mean IAT scores tend to show slightly stronger associations corresponding to the pairings of the combined block that is completed first (Nosek et al., 2005). To control for this effect, the order of the combined blocks was counterbalanced between participants as proposed by Nosek et al. (2007).

#### Post-experience questionnaire

After each exposure a 5-statement post-questionnaire was administered to assess subjective experience of participants (Table 2). A 7-point scale was used ranging from −3 to +3, with “0” indicating a neutral response on each question (with the scale varying from Strongly Disagree to Strongly Agree). More specifically, these questions were related to the strength of body ownership (MyBody, Mirror) and agency (Agency)—here we require that the levels of body ownership and agency are the same between the two conditions—while others served as control questions (Features, TwoBodies).

### Ethics

The study was approved by Comissió Bioètica of Universitat de Barcelona. Ethical considerations included informed consent, right to withdraw, confidentiality and sources of support, if required. Exclusion criteria were epilepsy, use of medication, recent consumption of alcohol, significant back or neck difficulties, intellectual disability and mental health difficulties (e.g., requiring medication), as well race other than Caucasian. Inclusion criteria were as reported in Section Participants. Participants were paid between €15 and €25 (Euros) for participating, according to the number of exposures to which they were assigned, or a pro-rata rate if they chose to withdraw (5€ at the end of the first session and the rest after the end of the last session). Following completion of the last phase, participants were debriefed with an explanation about the purpose of the study. They were also given the opportunity to leave their name and address if they wanted a short summary of the outcomes of the study. All participants were contacted via email 1 week after completion of the experiment to make sure there were not any undesired “after-effects” from exposure to the VR system.

## Results

### Experiment 1

**Figure 2:**
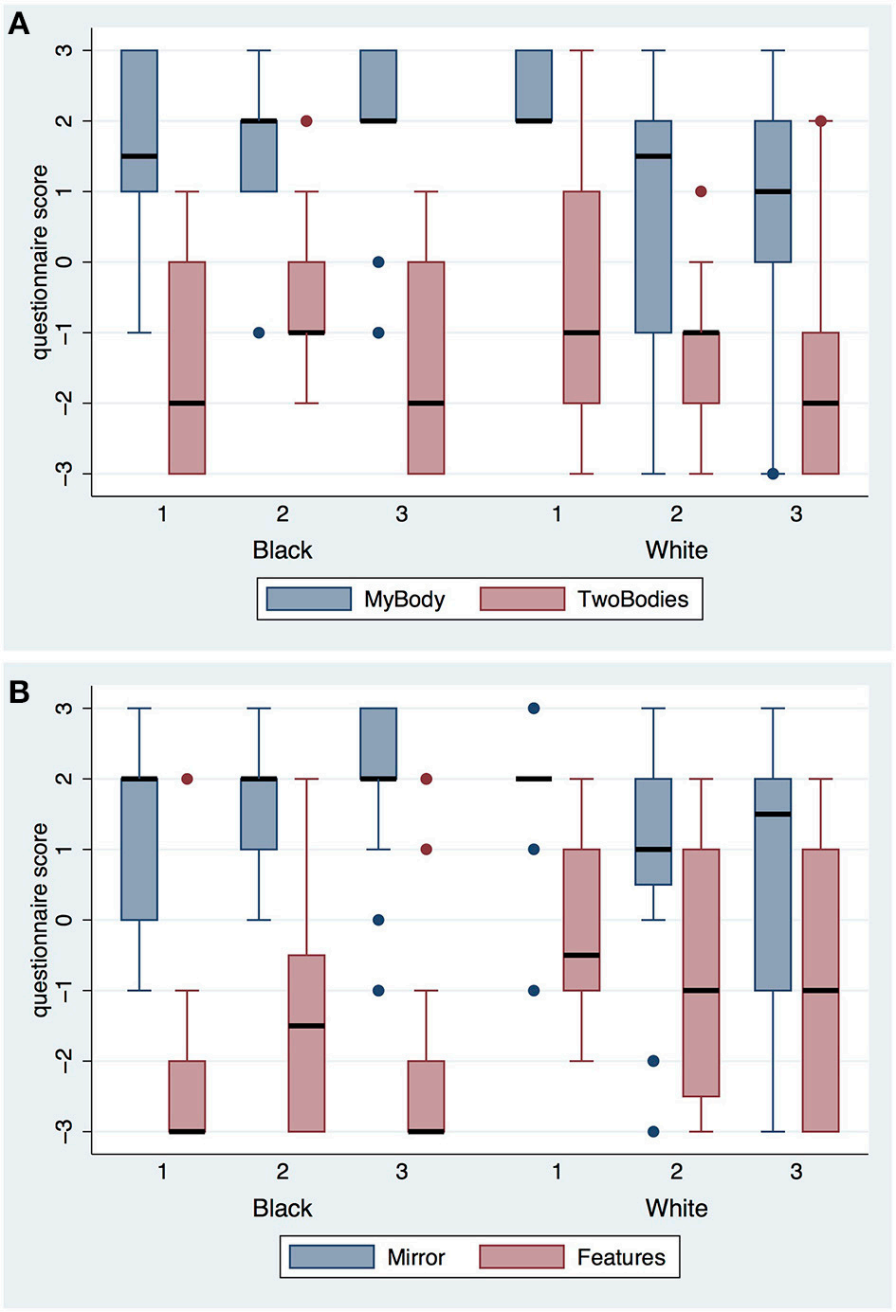
**Box plots of body ownership questions by Embodiment and Exposure (A)** for MyBody and TwoBodies **(B)** for Mirror and Features. The thick black horizontal lines are the medians, the boxes are the interquartile ranges, and the whiskers extend to ±1.5 × IQR, or the range. Individual points are outliers.

First we consider the questionnaire responses on body ownership and agency (Table 2). Figure 2 shows the scores on body ownership. The variable MyBody refers to the degree to which participants felt as if the body they saw when looking toward themselves was their body, TwoBodies refers to the extent to which they felt they had two bodies. Mirror refers to the body they saw in the mirror, and Features refers to the extent to which participants affirmed that the virtual body had physical features in common with themselves. TwoBodies is considered as a control question for MyBody and Mirror. All are on a scale from −3 to 3, where −3 means strongly disagree and +3 strongly agree. It is clear that participants tended to affirm the virtual body as their own, irrespective of skin color and irrespective of the number of exposures. On the other hand, they tended to disagree with the feeling of having two bodies. Comparing TwoBodies with MyBody and Mirror it can be seen that even the interquartile ranges of TwoBodies do not intersect those of MyBody and Mirror. Features is the lowest for the Black Embodiment and somewhat higher for the White Embodiment, in line with the fact that the White virtual body would have more in common with the bodies of the participants than the Black body. To test whether there is a difference in scores on Features between the Black and White body we carried out a mixed effects ordered logistic regression of Features on Embodiment × Exposure. Neither the interaction term (*P* > 0.38) nor Exposure as a main effect (*P* > 0.79) are significant, whereas eliminating Exposure we find that Embodiment has *z* = 2.86, *P* = 0.004. Hence the appearance in Figure 2B that Features in the White Embodiment is greater than in the Black Embodiment is supported.

Agency, referring to the extent to which participants affirmed that the virtual body's movements were their own (Table 2), had a median of 3 in all conditions with the interquartile range, and in most cases the range, no more than 1. This reflects that the real-time motion capture system worked well, since it was in fact the case that the virtual body was programmed to move in synchrony and correlation with real body movements.

**Figure 3:**
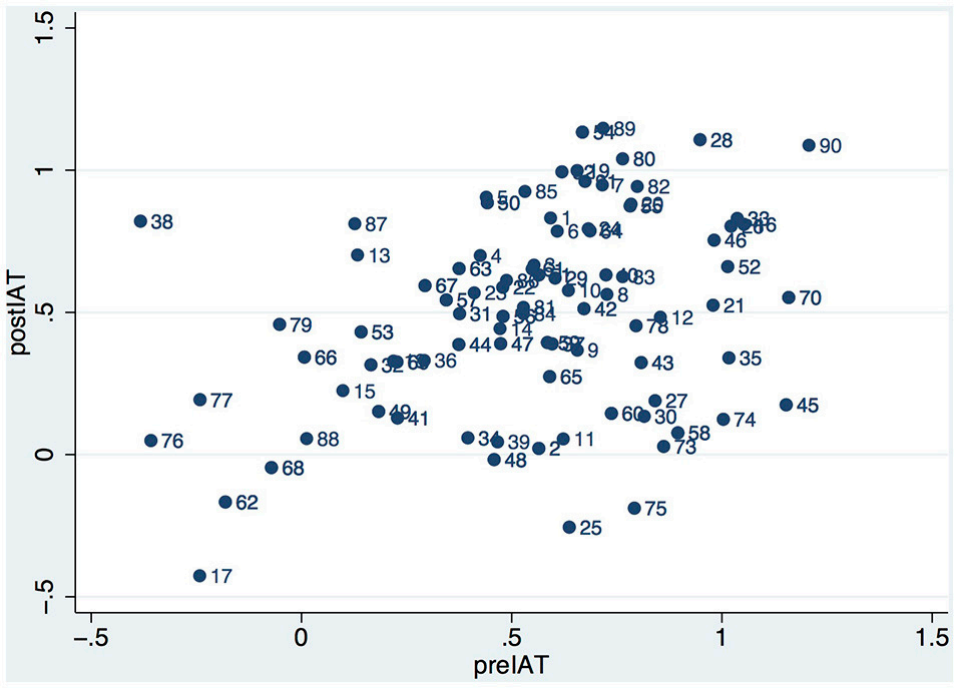
**Scatter diagram of postIAT by preIAT for all participants in Experiments 1 and 2**.

Figure 3 shows the plot of postIAT by preIAT across participants in both experiments. It is clear that there is one outlier (id 38), corresponding to a participant in Experiment 1 (Asian teacher) who had 2 exposures. This data point is excluded from subsequent analysis.

Recall that the racial bias IAT was measured for each person 1 week prior to their first virtual reality exposure. Lower values of IAT indicate less implicit bias against Black. For Experiment 1 preIAT has mean 0.59 ± 0.037 (SE) (*n* = 59). For those in the White embodied group the postIAT mean ± SE is 0.60 ± 0.055 (*n* = 30), and for those in the Black embodied group 0.36 ± 0.059 (*n* = 29). In line with previous results the Black embodiment appears to have reduced the degree of bias but not changed bias to non-bias.

**Figure 4:**
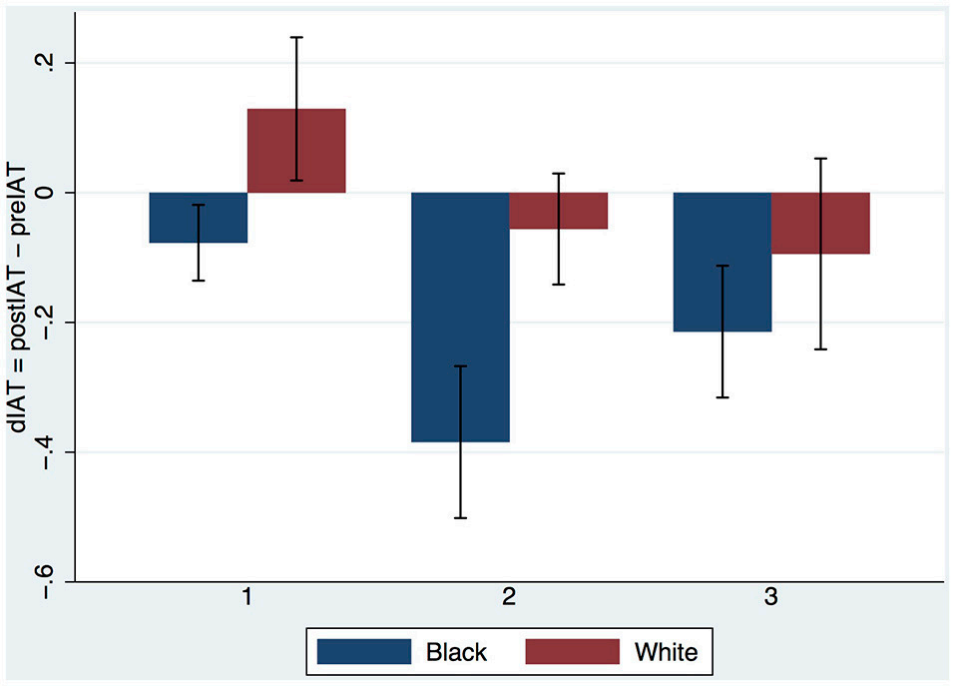
**Bar chart (means and standard errors) of dIAT by Embodiment (Black, White) and Exposures (1,2,3), Experiment 1**.

Figure 4 provides a more complete picture showing the means and standard errors of dIAT by the two factors. Recalling that the postIAT was measured 1 week after the final exposure what is interesting is that the results are quite similar to the single exposure experiment of Peck et al. (2013) where the postIAT was measured immediately after the exposure. In that earlier experiment we also found that the mean IAT increased for the White embodied group and decreased for the Black embodied group. We see the same here for those who had only 1 exposure. The number of exposures may have an influence and at each exposure level the decrease in IAT appears to be greater in the Black embodied group than the White.

Using dIAT as the response variable for ANOVA results in residual errors that are not compatible with normality whereas using postIAT as the response variable with preIAT as a covariate resolves this problem—although the results in both cases are very similar. For comparison purposes with the results of Peck et al. (2013) we provide the analysis using dIAT in Supplementary Text (#1). ANCOVA of postIAT on Embodiment × Exposures with preIAT as the covariate reveals no interaction (*P* > 0.58). Removing the interaction term we find that Embodiment has *F*_(1, 55)_ = 9.02, *P* = 0.004 (Partial η^2^ = 0.14), Exposures has *F*_(1, 55)_ = 1.96, *P* = 0.17 (Partial η^2^ = 0.03). The Overall *R*^2^ = 0.26. If we therefore remove Exposure then Embodiment has *F*_(1, 56)_ = 7.81, *P* = 0.004 (η^2^ = 0.14). The covariate preIAT of course always makes a major contribution in each of the models. For example, in the last model preIAT has *F*_(1, 56)_ = 7.81, *P* = 0.007, (Partial η^2^ = 0.12). Overall *R*^2^ = 0.24. The Shapiro-Wilk test for normality of the residuals results in *z* = 1.34, *P* = 0.09.

### Experiment 2

We were interested in why dIAT might decrease with increasing exposures, even if slightly, also for those in the White body. This could have occurred simply because prior experience with IAT tends to diminish the likelihood of biased responses (Greenwald and Nosek, 2001) and in this experiment each participant was administered the IAT twice. However, if this were the case then we would have expected those with only one exposure to show a decreased IAT, but in fact for those in the White Embodiment group it increases. Alternatively, the effect might occur because of the contact hypothesis (Pettigrew and Tropp, 2006; Van Bavel and Cunningham, 2009) which states that positive contact with non-involved other out-group members can generalize to reduction in bias to other groups. In our case the other out-group member was the Teacher, who was of Asian appearance.

Therefore, we carried out a second experiment under the same conditions as the first and with a different group of 30 participants, except that the Teacher was of European appearance, and the participants (10 per cell) were always embodied as White. If the contact hypothesis were operating, we would expect a reduction in IAT after exposure.

**Figure 5:**
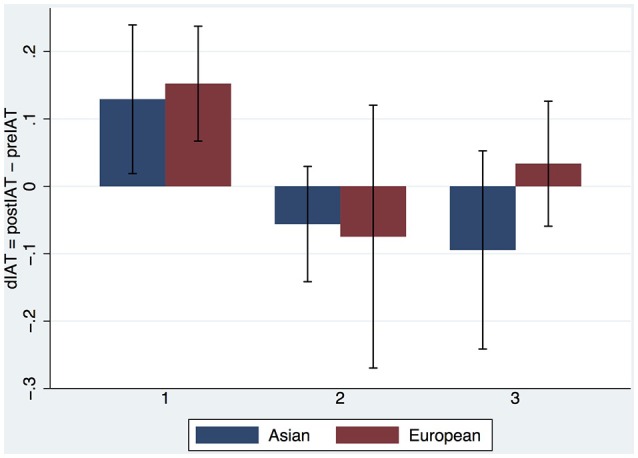
**Bar chart showing means and SEs of dIAT by Teacher and Exposures**.

The body ownership results are similar to those of Experiment 1 (Supplementary Text #2). Figure 5 shows the results for dIAT combining the Embodied White with the Asian Teacher from Experiment 1 and the new results from Experiment 2. We find again that the IAT increases after the first exposure with White Embodiment, repeating the result found by Peck et al. (2013) and in Experiment 1 above. There is a hint that indeed the Asian teacher might reduce the IAT more than the European if there is more than one exposure. However, ANCOVA of postIAT on Teacher × Exposures with preIAT as the covariate shows no interaction effect (*P* > 0.68) and no main effect of Teacher (*P* > 0.86). Eliminating the interaction term the main effect of Teacher is still not significant (*P* > 0.59) but Exposures has *P* = 0.037. Eliminating Teacher results in Exposures having significance level *P* = 0.036, *F*_(1, 57)_ = 4.60, *R*^2^ = 0.20. (Shapiro-Wilk test for normality of the residual errors results in *z* = 0.379, *P* = 0.35).

**Figure 6:**
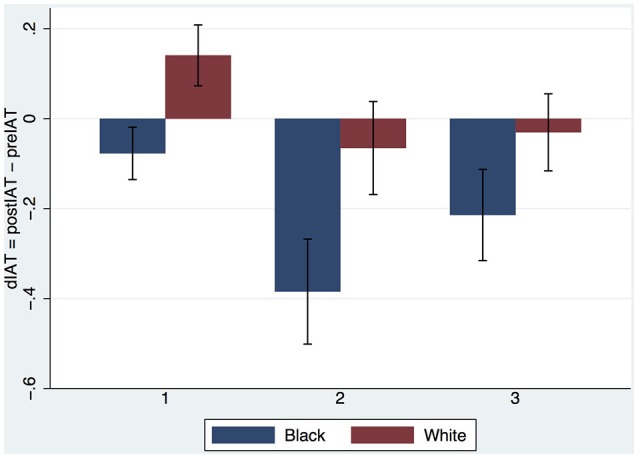
**Bar chart showing means and SEs of dIAT for all observations (***n*** = 89) by Embodiment and Exposures**.

Since Teacher has no effect we can pool all results of both experiments together and test amongst all *n* = 89 participants (recall that there is one deleted observation) for the effect of Embodiment and Exposures on postIAT with preIAT as a covariate. This is shown in Figure 6. ANCOVA of postIAT on Embodiment × Exposures with preIAT as covariate shows no interaction effect (*P* > 0.43). Eliminating the interaction term we find that Embodiment results in *F*_(1, 85)_ = 9.34, *P* = 0.003, Partial η^2^ = 0.10. For Exposures *F*_(1, 85)_ = 4.51, *P* = 0.036, Partial η^2^ = 0.05. (The Shapiro-Wilk test for normality results in *z* = 1.68, *P* = 0.046).

### Summary of findings

The evidence from Experiment 1 suggests that Embodiment in the Black body results in a reduction of implicit racial bias, even 1 week after the end of the experiment. However, the evidence for the influence of multiple exposures is more ambiguous. Figure 4 indicates that there may be some effect even if not statistically significant. The results from Experiment 2 support the conclusion that the Embodied Black condition does reduce implicit bias irrespective of the number of exposures. However, there is also evidence that bias also decreases with the number of exposures independently of the Embodiment factor (White or Black body). The evidence suggests that the Teacher (Asian, Caucasian) had no influence on bias.

## Discussion

### Body ownership

Body ownership through first person perspective (1PP) with respect to the virtual body that visually substitutes the real body is a long-standing result. For example, Petkova and Ehrsson (2008) used video cameras mounted on the head of a manikin looking down toward its body, but fed the video stream via a head-mounted display to participants. Provided they were looking down they would see the manikin body as substituting their own. Combined with synchronous visuotactile stimulation on the manikin and real body, so that participants saw something touching the manikin trunk while feeling this synchronously and correspondingly with touches on their body, there was an illusion of body ownership. When there was visuotactile asynchrony the illusion of ownership was significantly reduced. This was backed up by arousal responses (skin conductance) when the manikin body was attacked, but only in the visuotactile synchronous condition. Slater et al. (2010) used virtual reality to similar effect—when participants in a head-tracked head mounted display delivered VR and looked down toward their real body and in a virtual mirror they would see a life-sized virtual body, including the movements of the virtual head that were synchronous and in correspondence with their real head movements. There was a strong illusion of body ownership in the 1PP condition compared to a third person perspective (3PP) condition. Moreover, a strong heart-rate deceleration corresponded to seeing the virtual body attacked in the 1PP but not in the 3PP condition. Unlike Petkova and Ehrsson (2008) synchronous visuotactile stimulation was not necessary to achieve this effect. These two results are discussed by Maselli and Slater (2013), where it was found that 1PP was dominant, and the discrepancy between the two studies might be accounted for by the plastic look of the manikin in the first study, and the more realistic appearance of the virtual body in the second (i.e., with more human skin tones). Petkova et al. (2011) also support 1PP as a critical contributor to the ownership illusion.

In the current experiment we used visuomotor synchrony based on real-time motion capture data applied to the virtual body rather than visuotactile synchrony. Several studies have found strong ownership illusions using this method in combination with 1PP—for example (Banakou et al., 2013; Banakou and Slater, 2014). Kokkinara and Slater (2014) directly compared the effects of individual and combined influences of visuomotor and visuotactile synchrony, and found that visuomotor was more likely to lead to the illusion, but that in the case where both visuomotor and visuotactile were present the illusion could be equally broken by changing either one of them to asynchronous.

Finally, there is also the evidence suggesting that the appearance of the virtual human body does not play a role in the illusion. For example, in Slater et al. (2010) the participants were all men but virtually embodied as a young girl. In Kilteni et al. (2013) and Peck et al. (2013) all participants were light-skinned but the level of body ownership did not differ between a light-skinned, dark-skinned or even a purple body. In Normand et al. (2011) the participants were thin males, but had subjective ownership over a fat virtual body experienced from 1PP and with visuotactile synchrony. Most recently in Osimo et al. (2015) young male participants experienced a virtual body that was a scanned 3D digital copy of their own real body, and a virtual body that was much older depicting Sigmund Freud, in a within group design. Again there was no difference in the level of body ownership even when one virtual body looked very much like themselves, and the other was much older.

Blanke et al. (2015) argued that body consciousness requires integration of proprioception, top-down body-related visual information in peripersonal space, and embodiment. Our setup well integrates these, and is compatible with the multisensory integration approach discussed by Ehrsson (2012). Since there is visuomotor synchrony, proprioception is intimately connected with body-related visual events occurring in peripersonal space, with embodiment such that all sensory information about the virtual body is centered on the location of the real body. Putting this simply, and as we argued in Banakou and Slater (2014), in our whole lives whenever we look down we see our own body. Whenever (under normal healthy conditions) we move our limbs we feel them move (proprioception) and what we see moving are our own limbs. This history therefore provides overwhelming evidence that what we see in virtual reality under conditions of 1PP + visuomotor synchrony is our own body. The fact that it does not look like our body seems to be discounted in the brain's computation of body ownership, and in any case in terms of perception there is no “alternative hypothesis” since the virtual body is the only visible body in peripersonal space. Clark (2013) in a review of the predictive brain paradigm points out that the brain's perceptual system constructs models based on top-down connections that predict a version of the sensory stimuli it is receiving. Hence the model “this is my body” would certainly account for the multisensory information that the virtual body is both visually where the body should be, and integrates proprioception with intentional movement, and looks like a human body. However, it seems that the brain extends this model to include consequences of this being “my body” even when it does not look like it normally looks.

### Reducing implicit racial bias

The study has several findings in relation to implicit racial bias. The first is that the earlier results reported by Peck et al. (2013) have been reproduced taking into account those who had only one exposure. After one exposure for those embodied in the Black virtual body the mean implicit bias against Black decreases compared to those embodied in the White body. Second, the reduction is sustained for at least 1 week. Third, the number of exposures may have an effect independently of type of body, a point we will return to below.

It is important to note that the setting of the experiment was a benign one—a Teacher showing the participants various Tai Chi movements. In contrast, the finding of Groom et al. (2009) stands out as the one case where embodiment of White participants in a Black virtual body resulted in an increase in implicit racial bias. Although there are many technical differences between the setup in that experiment and ours, as detailed by Peck et al. (2013), and there was no notion of body ownership, the fundamental difference is that the Groom study included a negative social setting (a job interview) that in itself was related to racial bias. In the present study, unlike that of Peck et al. (2013) there was also a social setting—but it was a benign one, unrelated to the issue of racial bias.

Amongst the least successful 17 interventions for the reduction of bias discussed by Lai et al. (2014) were those that employed perspective taking. Superficially perspective taking is, amongst the methods considered, the closest to the technique of embodiment. Participants were required to imagine that they were the person shown in a picture and describe how they might experience an event as that person, their emotions, thoughts and feelings, based on the method described by Ames et al. (2008). Thus, with the subject of the experiment being White and the person shown in the picture being Black, this would be like an imaginal equivalent of embodiment. However, the critical point is that it is imaginal, whereas virtual embodiment is direct, leading to a perceptual illusion of body ownership. Moreover, in the case of virtual embodiment participants are not required to imagine anything or think about how it would be to be that person, they simply see the virtual body that moves in correspondence with their own movements, both by looking toward it and in a mirror. Participants only have to experience, they do not have to imagine. Moreover, unlike the studies reported by Lai et al. (2016) the evidence suggests that the embodiment intervention results in a sustained reduction of implicit bias that lasts at least 1 week. We discuss some possible reasons why in the next section.

### The IAT

One way to think about an IAT is that it samples for any individual their statistical associations between categories. If over a long period a person has been subject to information that X is associated with Y but not Z and they are given X with two cues Y and Z there is a higher probability that they will choose Y at a greater speed than they would choose Z in a forced choice. A racial bias IAT ultimately tests the strength of such associations. We contend that in most Western countries there has been a greater preponderance in the media of negative associations with the concept of “Black” people compared to “White,” and the racial IAT reflects this in spite of the explicit attitudes of people, so that there is a dissociation between the implicit and explicit bias (Greenwald and Krieger, 2006). Indeed in the explicit racial attitudes test in Peck et al. (2013) there was no evidence of racial bias, even though the pre-experiment IAT showed implicit bias. However, when it comes to discriminatory behavior the IAT results have better predictive power for social interaction than explicit measures (Greenwald et al., 2009)—for example, with respect to eye contact, proxemics, and hiring practice (Ziegert and Hanges, 2005; Rooth, 2010). Even though the use and interpretation of the IAT may be controversial there is significant evidence of its explanatory and predictive power (Jost et al., 2009).

Now based on this model of how the IAT may work we consider how it can be possible that relatively short exposures of embodiment in a Black virtual body can impact and reduce implicit bias. One answer may be that body ownership and agency over a virtual body is more than a superficial illusion, that it goes beyond the perceptual to influence cognitive processing. We argued in Banakou et al. (2013) and Llobera et al. (2013) that a fundamental mechanism may be through the postulated “cortical body matrix” (Moseley et al., 2012), that maintains a multi-sensory representation of the space immediately around the body in a body-centered reference frame. The system is responsible for homeostatic regulation of the body, and for dynamically reconstructing the body representation moment to moment based on current multisensory information. Moreover, we propose that it also maintains an overall consistency between the multifaceted aspects of self (personality, attitudes, behaviors) and the body representation. In other words our suggestion is that when the body changes not only are there updates to the multisensory representation of peripersonal space but also there are corresponding psychological updates. For example, when adults are embodied in a child body not only do they overestimate object sizes but they self-identify more with child-like attributes in an IAT classifying self with adult or child-like attributes (Banakou et al., 2013). We can view IAT changes as direct evidence of this idea, that changing the body apparently leads to changes in implicit attitudes. This is not a process whereby participants in any way *believe* that their body has changed, nor might they explicitly say that their attitude is now different, but it is a process that occurs below the threshold of consciousness. We can say that as well as body ownership over a different body leading to changes in implicit attitudes, the documented changes in implicit attitudes are a very strong signal that in fact there has been a change in body ownership.

Now as we argued the IAT is simply a statistical measure of association between categories for any individual, for example, based on a lifetime of statistical associations from the social environment. We can think of this as there being a current joint probability distribution over the sets of categories, and the IAT is based on sampling this distribution. Now thinking of this in Bayesian terms the process of embodiment, where e.g., the White person now has a Black body, provides a strongly weighted piece of new evidence that leads to an update of this probability distribution. The vast amount of statistical evidence that made up the probability distribution is for most people based on impersonal evidence. It is just picked up from everyday commentary, for example, in the media. Now though there is a new piece of “evidence” of critical importance—“I” have a Black skin, and “I” in turn carries with it a whole set of associations with the positively/negatively valenced categories that are likely to be very different from the associations with the out-group. This leads to a new probability distribution—as if the new embodiment information disrupts the previous associations between categories. The one piece of new evidence is a critical one—since it is about the self. Hence it could be argued that the changes in IAT are produced by a disruption of the standard associations between “Black” and “negative”—based on the new observation that “I can be Black.” In the terminology of the opening paragraph of this section X (Black) is typically associated with Y (a set of negative attributes) but not Z (a set of positive attributes), so that in rapid sampling of associations with X there is higher probability of choosing elements from Y instead of Z. However, the embodiment of Self as Black can change the associations with X because X has now been identified with Self, which in turn will have its own distribution of probabilities over elements in Y and Z. Hence sampling the associations with X after embodiment is, in this explanation, likely to lead to a different outcome than prior to embodiment. However, this argument relies on the participant being more likely to have positive (Z) than negative (Y) associations with Self. This leads to the testable hypothesis that the reduction in IAT should be more likely to occur for individuals with higher self-esteem than those with lower self-esteem. We do not have such data in this experimental study, but this is left as further work as a way to test this model.

From our current results it seems that just one exposure is already sufficient to disrupt the probability distribution in this model. This argument is similar to that of Maister et al. (2015): that similarity between appearance of the self (as transformed during body ownership) and the out-group results in the disruption of associations between the out-group and negative valence items, and substituted by positive associations with the self.

### The IAT and saliency

Treating IAT as measuring associations though is only one way (even if the dominant one) for understanding how this measure works. Rothermund and Wentura (2004) introduced an alternate figure-ground explanation, where the most salient aspects of the target tend to be associated with the most salient attributes, independently of whether there are psychological associations between the two categories. For example, suppose the target categories are the musical instruments sitars and pianos, and the attributes are words with positive and negative valence. The explanation supposes that sitars are more salient, being the more unusual in most countries, appearing as a figure against the background of the more common pianos. Similarly, unpleasant words stand out more against the background of pleasant or neutral words. Hence subjects find it easier to quickly associate two salient categories together, even though there may be no intrinsic psychological association between sitars and negative words. In a series of experiments Rothermund and Wentura (2004) provided strong evidence that this explanation is viable—although the two different explanations, associative and figure-ground, are not mutually exclusive.

In our experiment it could be argued that the racial categorisation “Black” is more salient than White, and therefore is more likely to be associated with salient negative attributes compared to positive. In this explanation embodiment of a White person as Black changes the ground, so that Black is no longer salient. However, this seems unlikely as an explanation for these particular results. Participants had short embodiment exposures and otherwise during the time of the experimental period of course lived their normal lives in society where “Black” would be more salient than “White.” Also we would expect that more exposures as Black might make “Black” less salient, but the number of exposures operated independently of the type of embodiment. However, in the associations based explanation a single embodiment exposure as “Black” in itself provides evidence “I can be Black” and thus disrupt associations between positively/negatively valenced attributes (since these are now confounded with associations that apply to the self).

### The number of exposures

The results suggest that the number of exposures may be associated with a decrease in implicit bias irrespective of the type of embodiment. The explanation that this may be due to the contact hypothesis was not borne out by Experiment 2. Apart from simply being in VR the only other invariant across the two embodiment groups was that all practiced Tai Chi movements as part of the experiment. Tai Chi has been shown to have positive psychological benefits including a reduction of stress and anxiety, and more generally a number of positive health advantages—see the meta studies by Sandlund and Norlander (2000) and Jahnke et al. (2010). As noted by Sandlund and Norlander (2000) Tai Chi has elements in common with mindfulness meditation. Moreover, as reported by Lueke and Gibson (2014) mindfulness meditation reduces implicit racial and age bias. In that study participants listened to a 10 min mindfulness or a control recording. Those in the mindfulness group showed statistically significant but moderately less implicit bias than the control group. Similarly, and with more striking results Lueke and Gibson (2016) showed that a group that received a brief mindfulness meditation exposure exhibited less behavioral racial bias than control groups. Also in the context of prejudice against persons with disabilities, Schimchowitsch and Rohmer (2016) also showed moderate effects in the reduction of bias in a group that practiced yoga and mediation compared to a control group that did not. In our study the greater the number of exposures that participants had the greater their exposure to Tai Chi, with each Tai Chi session lasting for 10 min. Of course we cannot know whether or how much they entered any kind of meditative state, or whether they thought any more about their experience outside of the experimental sessions. It remains an intriguing possibility though that simply their exposure to Tai Chi may have influenced their level of implicit bias. We leave this as an open question for further research.

## Conclusions

Returning to the questions that this paper addressed our overall findings are that: first, body ownership over a differently raced body has again been shown to occur, suggesting that ownership is not contingent on the appearance of the virtual body. Second, there is further evidence that embodiment of White people in a dark-skinned virtual body does diminish their implicit racial bias. Third, this diminution lasts at least 1 week after the end of the exposure. Finally, one exposure is sufficient to observe this effect. Further replication studies would be needed to further support the last point. Moreover, it is important to note that the experiment involved only female participants. While there is some evidence that there is greater implicit racial bias amongst females compared to males (but greater explicit bias of males compared to females) (Ekehammar et al., 2003), further replication studies should address this issue.

## Data accessibility

The datasets supporting this article have been uploaded as part of the Supplementary Material.

## Author contributions

MS designed the experiment with the help of DB. The computer programming was carried out by DB and PH. The experiment was run by DB, the statistical analysis was carried out by MS, the paper was written by MS and DB, and checked by all authors. PH contributed to this work while a student on the MSc Computer Graphics, Vision & Imaging at UCL, during which time he carried out the project at the Event Lab, University of Barcelona.

## Funding

This research was funded under the European Seventh Framework Program, Future and Emerging Technologies (FET) project VERE (#257695). DB was supported by the FI-DGR 2014 Agència de Gestió d'Ajuts Universitaris i de Recerca, Generalitat de Catalunya, Ajuts per a la contractació de personal investigador novell.

### Conflict of interest statement

The authors declare that the research was conducted in the absence of any commercial or financial relationships that could be construed as a potential conflict of interest.

## Footnotes

^1^A higher resolution version can be seen on https://youtu.be/RcBJ1sCPv_I

^2^http://www.naturalpoint.com/optitrack/products/motive/body/

^3^http://usa.autodesk.com/3ds-max/

^4^https://charactergenerator.autodesk.com

^5^http://unity3d.com

^6^http://abranimations.com/new-mocap-tai-chi-animation/

^7^http://www.autodesk.com/products/motionbuilder/overview

## Supplementary material

Supplementary Video S1 **A video illustrating the various conditions of the experiment**.
