# Supplementary material for: Virtual Embodiment of White People in a Black Virtual Body Leads to a Sustained Reduction in Their Implicit Racial Bias
Source: Front Hum Neurosci. 2016 Nov 29;10:601. doi: 10.3389/fnhum.2016.00601 (PMC5126081; doi:10.3389/fnhum.2016.00601)
Supplement: Supplementary file 3 [file Presentation1.PDF]

Virtual Embodiment of White People in a Black Virtual Body Leads to a Sustained Reduction in their Implicit Racial Bias

Domna Banakou, Parasuram Dora H, Mel Slater

Supplementary Text

1. Analysis using dIAT as the Response Variable

As noted in the paper using dIAT = postIAT - preIAT results in linear models that do not satisfy the requirement that the residual errors approximately follow a normal distribution. We present the results in any case.

1.1 Experiment 1

ANCOVA of dIAT on Embodiment × Exposures reveals no significant interaction ($P > 0.68$). Removing the interaction term we find that Embodiment has $F(1,56) = 5.93$, $P = 0.018$ (Partial $\eta^2 = 0.10$), exposures has $F(1,56) = 2.89$, $P = 0.095$ (Partial $\eta^2 = 0.05$). Overall $R^2 = 0.14$. The Shapiro-Wilk test for normality of the residual errors results in $P = 0.037$. Removing Exposures, Embodiment has $F(1,57) = 5.74$, $P = 0.02$ ($R^2 = 0.09$). In this case the Shapiro-Wilk statistic gives $P = 0.013$ for the null hypothesis that the residual errors are normally distributed.

1.2 Experiment 2

ANCOVA of dIAT on Teacher × Exposures again has no significant interaction ($P > 0.67$). Removing the interaction term neither Teacher ($P > 0.66$) nor Exposures ($P > 0.17$) main effects approach significance. However, the residual errors of the fit are not compatible with normality (Shapiro-Wilk $P = 0.003$).

1.3 Pooled results

Since neither exposures nor Teacher have any effect we can pool all results together and test amongst all $n = 89$ participants (recalling one deleted observation). ANCOVA of dIAT on Embodiment × Exposures shows the interaction term with $P > 0.86$. Eliminating the interaction results in Embodiment: $F(1,86) = 8.17$, $P = 0.005$, $\eta^2 = 0.09$; Exposures: $F(1,86) = 2.90$, $P = 0.092$, $\eta^2 = 0.05$. However, the Shapiro-Wilk test for normality of the residual errors has $P = 0.0007$.

2. Body ownership and agency for experiment 2

Figure S1 shows the body ownership and control questions, equivalent to Figure 2 in the main text. Agency had a median of 3 with interquartile range 1 for all three Exposures.

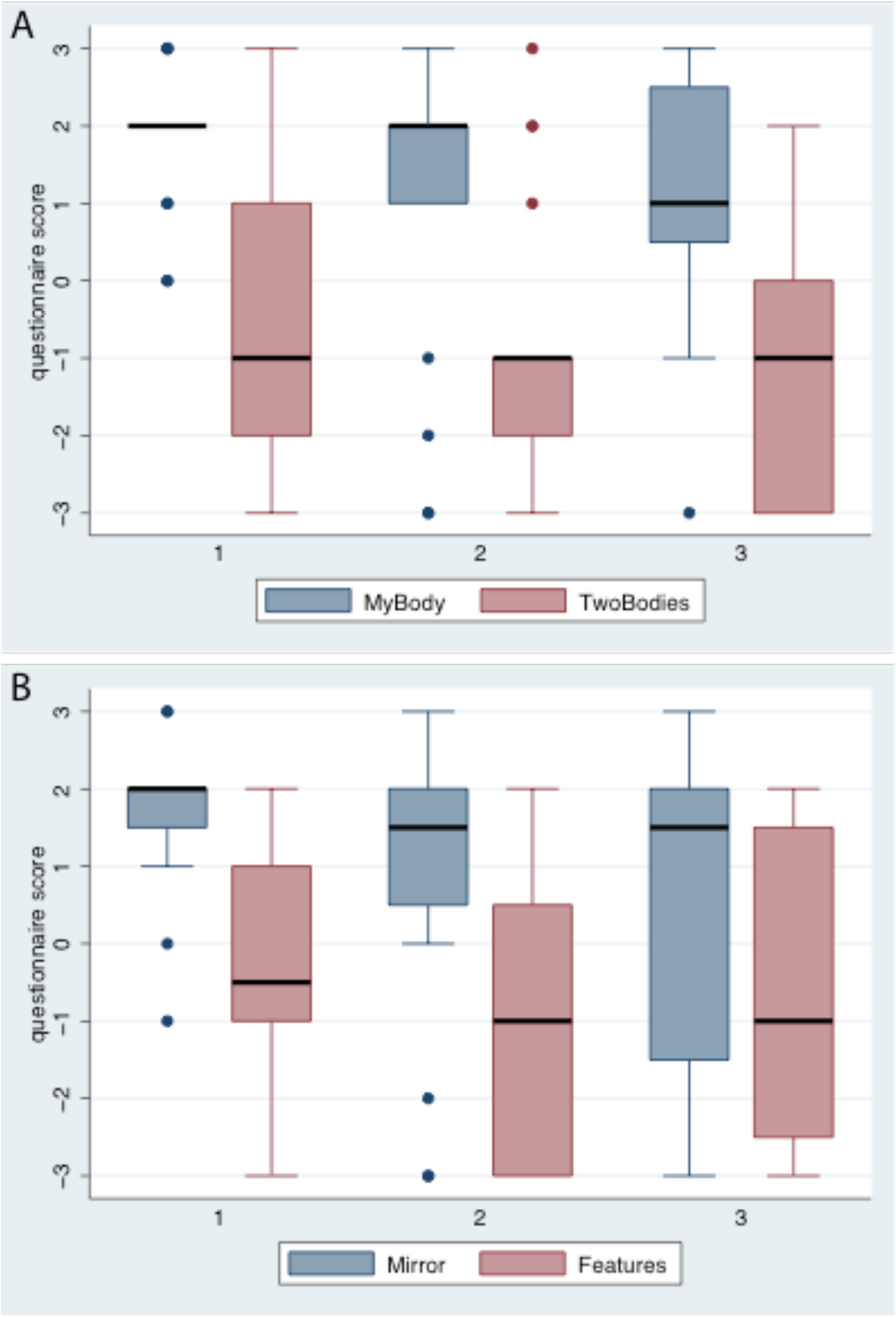

Figure S1 - Box plots of body ownership questions by Exposure in Experiment 2 (European Caucasian Teacher) (A) for MyBody and TwoBodies (B) for Mirror and Features. The thick black horizontal lines are the medians, the boxes are the interquartile ranges, and the whiskers extend to ±1.5×IQR, or the range. Individual points are outliers.
